# Supplementary material for: The interactome of intact mitochondria by cross-linking mass spectrometry provides evidence for coexisting respiratory supercomplexes
Source: Mol Cell Proteomics. 2017 Dec 8;17(2):216–32. doi: 10.1074/mcp.RA117.000470 (PMC5795388; doi:10.1074/mcp.RA117.000470)
Supplement: Supplemental Data [file supp_RA117.000470_133922_0_supp_23978_fzffwf.zip › spectra_annotation/mito_DR_spectra_annotation/214-1-14-1-14-1.pdf]

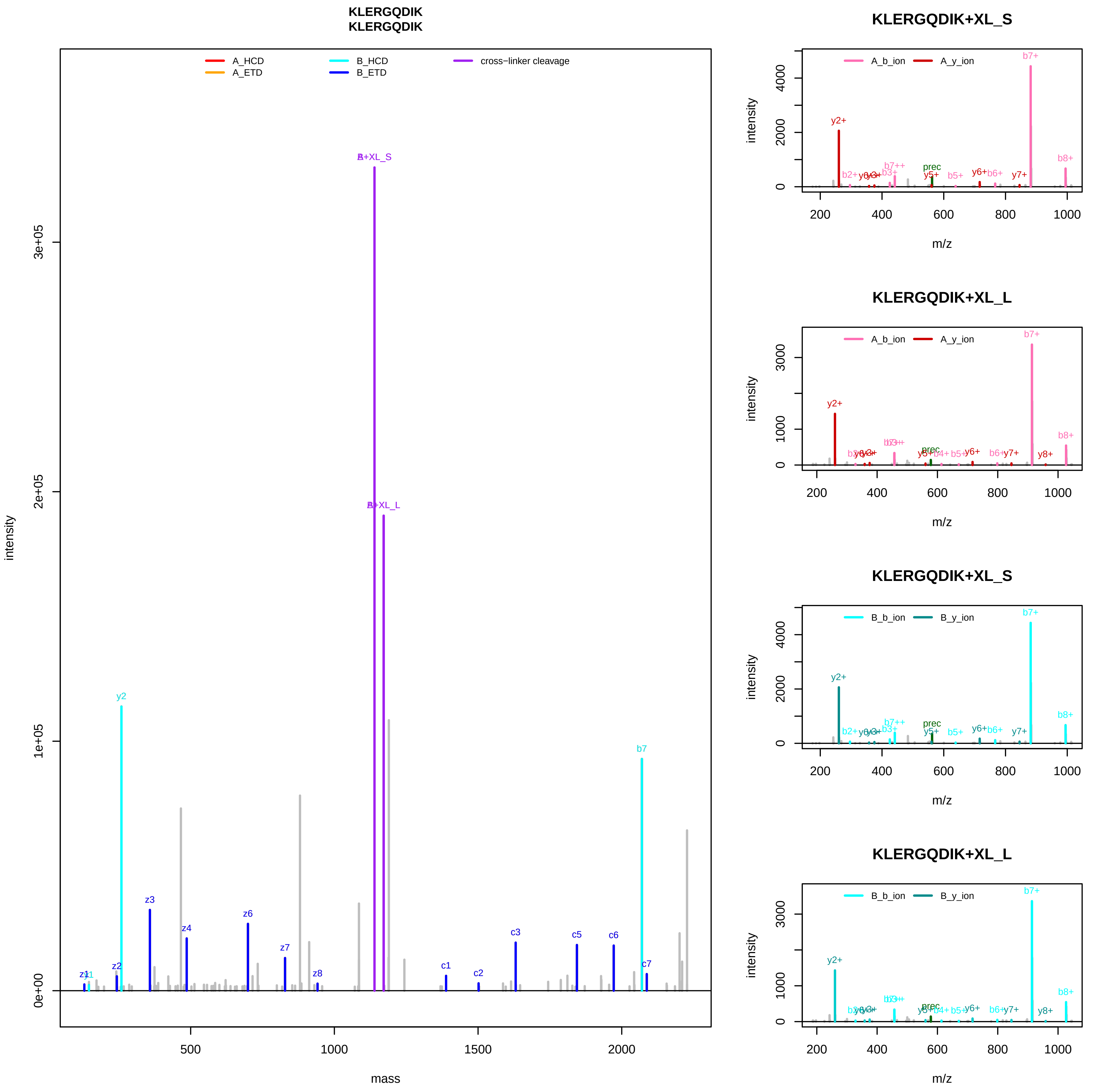
KLERGQDIK
KLERGQDIK
A_HCD
A_ETD
B_HCD
B_ETD
cross−linker cleavage
intensity
mass
B+XL_S
B+XL_L
y2
z3
z4
z6
z7
z8
z1
y1
z2
c1
c2
c3
c5
c6
c7
b7
0e+00
1e+05
2e+05
3e+05
500
1000
1500
2000
KLERGQDIK+XL_S
A_b_ion
A_y_ion
m/z
KLERGQDIK+XL_L
KLERGQDIK+XL_S
B_b_ion
B_y_ion
KLERGQDIK+XL_L
b7+
b8+
y2+
prec
y5+
y6+
y7+
b5+
b6+
b3+
b7++
b2+
y8+
b4+
